# Does rejection have a role in lymphocele formation post renal transplantation? A single centre experience

**DOI:** 10.4103/0970-1591.65385

**Published:** 2010

**Authors:** Muthu Veeramani, Sashikant Mishra, Abraham Kurien, Arvind Ganpule, Ravindra Sabnis, Mahesh Desai

**Affiliations:** Department of Urology, Muljibhai Patel Urological Hospital, Nadiad, Gujarat, India

**Keywords:** Lymphocele, rejection, renal transplantation

## Abstract

**Aim:**

To assess the relation of acute rejection with respect to lymphocele incidence and determine the effect of lymphocele with graft survival.

**Methods:**

The paper is a singlecenter retrospective data review of renal transplant recipients from 1980 to 2007. A total of 1700 patients received kidneys from live donation, and 9 patients received from cadaver donor. The standard transplant technique was performed in all. Lymphocele incidence, demography, relation to rejection episodes, type of immunosuppression, and management options were studied. Univariate analysis was performed to assess the role of rejection to lymphocele formation.

**Results:**

47 (35 males and 12 females) patients had symptomatic lymphocele in the post-transplant period. 51% of the lymphocele patients had history of rejection as compared to overall rejection rate of 20% (*P* = 0.009). 4 (7.2%) had at least 1 rejection and 19 (40.4%) had more than one rejection episodes. All 47 patients required aspiration. Of the 14 patients who did not settle with a maximum of two aspirations underwent marsupilization (5 open and 9 laparoscopic). 1, 5, and 10 year graft survival of overall transplant recipient and post-transplant lymphocele patients was 86.54%, 82.41% and 76.36% vs. 86.44%, 81.2% and 68.14%, respectively.

**Conclusion:**

Acute rejection episodes were associated with statistically increased risk of lymphocele. There was no adverse outcome of graft with lymphocele formation after rejection episodes with respect to the overall graft survival.

## INTRODUCTION

Lymphocele following transplantation is not an uncommon problem. It has been reported in the literature in the range from 0.6% to 18%[1–3] Multiple risk factors have been described in the literature for the development of lymphocele. Animal studies have shown that there is 20- to 50-fold increase of lymph flow during rejection episodes. There is a very few literature support to provide evidence that rejection is a possible cause for lymphocele. To establish the role of acute rejection with respect to lymphocele formation requires a well-controlled prospective study. So far, there are only retrospective data to support the hypothesis. We performed a univariate analysis of rejection episodes with respect to the incidence of lymphocele formation.

## MATERIALS AND METHODS

After the approval of the institutional review board, the case records of the transplant patients treated for symptomatic lymphocele in our institute were analyzed. Between 1980 and 2007, 1700 live renal transplantations were carried out in our institute. 20% of them received kidneys from other than related, nine from deceased donor kidneys and the rest from related donors. During the initial period, open donor nephrectomy was performed but from 2002 onward kidney retrieval was through the laparoscopic method. Renal transplantation was done as per the standard technique. After ligating the lymphatics over the iliac vessels, in majority of cases, right iliac fossa transplantation was done. Ureter was reimplanted to the dome of the bladder by the modified Lich Gregoir method. Post-operatively, the patients were monitored for lymphocele by routine abdominal ultrasonography. As per the institute protocol, recipients received immunosuppression, which was either cyclosporine, azothioprine, prednisolone (CAP) or tacrolimus, azothioprine, prednisolone (TAP). There were no patients on mTOR inhibitors.

Patients presenting with symptomatic lymphocele were reviewed by their demographic pattern and clinical manifestations. Lymphocele was defined as the presence of a peri-renal fluid collection with a diameter greater than 5 cm, diagnosed after the first postoperative week. It was classified as symptomatic when associated with local and/or systemic signs and symptoms. Since the asymptomatic lymphocele data were not recorded in the case sheets of the patients, it was not included as study variable. Incidence of rejection episodes in the lymphocele group, number of rejection episodes, and type of immunosuppressant therapy were evaluated. Rejection episodes were confirmed by a renal graft biopsy after which they received anti-rejection therapy. Initially, all symptomatic patients were treated with two attempts of sonography-guided aspiration and injection of sclerosant. Patients who had a recurrence after initial aspiration were treated with either open or laparoscopic marsupialization.

Statistical analysis was performed with Student's *t*-test to establish the level of significance using the SPS software 15. *P* value <0.05 was considered statistically significant. Kaplan Mayer survival curve was used to assess the graft survival.

## RESULTS

**Figure 1:**
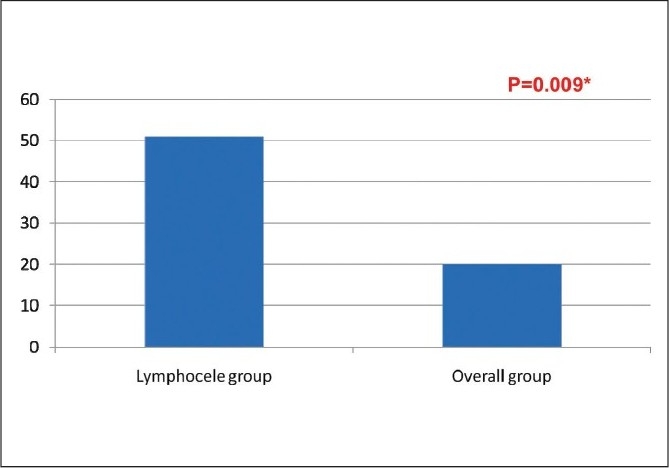
Rejection episodes in the lymphocele group and overall incidence

**Figure 2:**
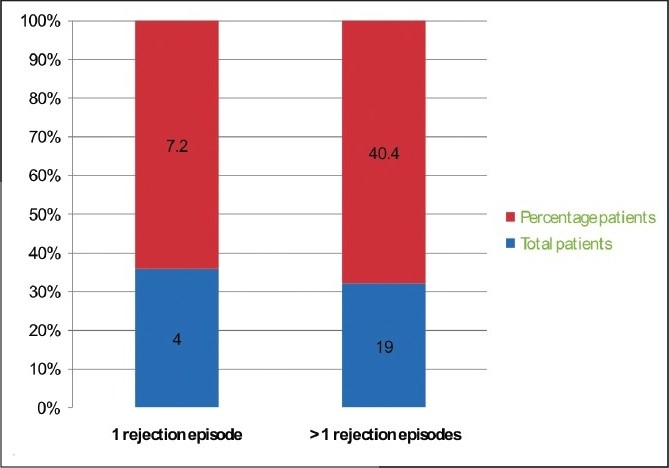
Number of rejection episodes in the lymphocele group.

Of the 1709 renal transplantations, 47 patients presented with symptomatic lymphocele. The clinical presentation of the patients is as in Table 1. There were 35 (74%) males and 12 (26%) females. Biopsy-proven rejection episodes in the overall transplants (n=1709) were 340 (20%). All cases studied had rejection episodes preceding lymphocele formation. In the symptomatic lymphocele group (47) the incidence of rejection episodes were 23 (50%) [Figure 1] and more than 40% (19) patients had more than one rejection episodes [Figure 2]. The technique of handling the lymphatics was same during the years in the recipient side. On the donor side, the open donor nephrectomy was replaced by laparoscopic donor nephrectomy. The incidence of lymphocele remained the same, inspite of other complications reducing over the period of time. The breakup of lymphocele incidence was 15 during first 500, 14 during the next 500, and 18 during the last 700 renal transplant procedures.

**Table 1:** Clinical presentation

Presentation	Incidence (n, %)
Rising creatinine	34 (72.3)
Edema of the ipsilateral thigh and genitalia	16 (34)
Hypertension	11 (23.4)
Suprapubicpressure	11 (23.4)
Urinary frequency	6 (12.7)
Mass adjacent to the allograft	1 (2.1)
Oliguria	2 (4.2)

**Figure 3:**
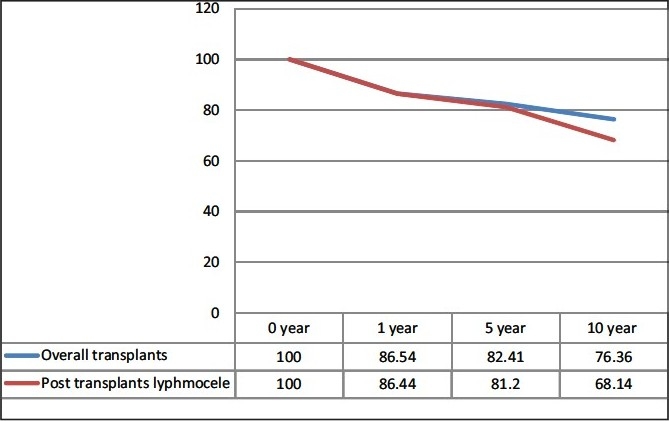
Graft survival in the lymphocele group with overall transplants

Overall graft survival and graft survival in the lymphocele group at 5 and 10 year was 82.4% vs. 81.2% and 76.36% vs. 68.14%, respectively [Figure 3].

19 symptomatic patients required marsupialization; 9 treated with open and 10 treated with the laparoscopic method. Two patients in each group had recurrence after marsupialization. Association of the lymphocele in the rejection group was significant (*P* = 0.009).

## DISCUSSION

The important source of lymph during renal transplantations is perivascularlymphatics of the recipient and the donor allograft lymphatics. Normally these sources can be blocked by ligation of the lymphatic channels during the surgery.[4] Cellular rejection of the kidney allograft has been described as a possible causal factor of lymphocele. This immunological phenomenon leads to an intense local inflammatory process and an increase in regional lymph flow. Fluid collection post-transplant is identified in many patients but majority undergo spontaneous resolution. Since, majority are small, most of them resolve unnoticed. Most of the collections are small and aymptomatic. It would be interesting to study the association of asymptomatic lymphocele converting into symptomatic once the rejection episodes occur. However, being a retrospective study, this association was not looked into. Symptomatic lymphoceles are of much less frequency but are easily recognized and diagnosed. The ocurrence of rejection increases 25- to 75-fold the risk of symptomatic and asymptomatic lymphoceles, respectively.[5]

The exact source of lymph production during rejection remains to be discerned. A possible mechanism to explain the increased flow of lymph from the kidney during cellular rejection was demonstrated by Pedersen and Moris.[6] These authors used a sheep model in which the kidney was implanted in the neck of the animals. They recorded the flow of the effluent after cannulating lymph ducts of the graft. A 20- to 50-fold increase in flow was observed during rejection. The fact that lymphoceles develop long after the operation despite meticulous ligation of the severed recipient lymphatics support the hypothesis that renal allograft lymphatics are primarily involved in the pathophysiology. Khauli *et al*.[7] analyzed the risk factors involved in the development of lymphatics following renal transplantation. In their study of 118 patients, 36% had fluid collection and only 22% of them required therapy. They found only rejection to be the variable significantly associated with the development of lymphoceles on multivariate analysis using logistic regression analysis. Boedker *et al*, however, could not demonstrate any statistical difference in the number of rejection episodes between patients with and without lymphoceles.[8]

## CONCLUSION

We conclude that there is a statistically significant increased incidence of lymphocele in patients with acute rejection episodes. On long-term follow up, treated symptomatic lymphocele does not have much impact on the graft survival when compare to the overall group. A prospective well designed study with lesser variables would give us a better answer and is strongly recommended for the future.

## References

[1] Shokeir AA, el-Diasty TA, Ghoneim MA (1993). Percutaneous treatment of lymphocele in renal transplant recipients. J Endourol.

[2] Dubeaux VT, Oliveira RM, Moura VJ, Pereira JM, Henriques FP (2004). Assessment of lymphocele incidence following 450 renal transplantations. Int Braz J Urol.

[3] Samhan M, Al-Mousawi M (2006). Lymphocele following renal transplantation. Saudi J Kidney Dis Transpl.

[4] Hamza A, Fischer K, Koch E, Wicht A, Zacharias M, Loertzer H (2006). Diagnostics and therapy of lymphoceles after kidney transplantation. Transplant Proc.

[5] Khauli RB, Stoff JS (1993). Post transplant lymphoceles: A critical look into the risk factors, pathopysiology and management. J Urol.

[6] Pederson NC, Morris B (1970). The role of lymphatic system in the rejection of homografts: A study of lymph from renal transplants. J Exp Med.

[7] Khauli RB, Mosenthal AC, Caushaj PF (1992). Treatment of lymphocele and lymphatic fistula following renal transplantation by laparoscopic peritoneal window. J Urol.

[8] Boedkar A, Roikjaer O, Rasmussen F, Loekkegaard H (1990). Lymphocele following renal transplantation: A clinical study. Transplant Proc.

